# Plant seedling growth and soil respiration responses to seasonal United Kingdom seaweed wrack extracts

**DOI:** 10.1007/s10811-025-03664-0

**Published:** 2025-10-09

**Authors:** Jose G. Gutierrez Higa, Adetunji Alex Adekanmbi, Samantha Kehoe, Tom Sizmur, Aaron Brown, Jessica M. M. Adams

**Affiliations:** 1 Institute of Biological, Environmental and Rural Sciences, Aberystwyth University, Gogerddan, Aberystwyth, SY23 3EE United Kingdom; 2 Department of Geography and Environmental Science, University of Reading, Whiteknights, Reading, Berkshire, RG6 6AH United Kingdom; 3 School of Chemical and Process Engineering, University of Leeds, Leeds, LS2 9JT United Kingdom

**Keywords:** *Ascophyllum nodosum*, Biostimulant, Biofertilizer, *Fucus*, Seaweed, Soil amendment

## Abstract

Macroalgal extracts offer an alternative option to increase crop yields and plant performance whilst reducing reliance on inorganic chemical fertilisers. Macroalgae have high concentrations of bioactive compounds capable of enhancing crop growth, stress tolerance and pest resistance. This study investigated whether seasonal variation in the chemical composition of three brown seaweeds *Ascophyllum nodosum*, *Fucus serratus* and *Fucus vesiculosus* affected plant growth in *Arabidopsis thaliana*, *Beta vulgaris* and *Lactuca sativa* through different extract concentrations. Crops were treated with 1:20, 1:50 and 1:100 dilutions from macroalgal extracts and compared to controls tap water, deionised water and one commercial macroalgae-based fertiliser made from *A. nodosum*. Dry weight assessment results revealed that moderately diluted dosages had better effects on plant growth than concentrated dosages, showing that the most suitable concentrations of all macroalgae extracts are 1:50 for *Arabidopsis*; no trend was detected in *B. vulgaris* or *L. sativa* growth. Overall, there were peaks of increased plant growth when treated with extracts harvested in June and August, which correlates with fertility peaks in commercial brown macroalgae in the wild. These results suggest that the optimal harvest for *A. nodosum*, *F. serratus* and *F. vesiculosus* for plant stimulant products should occur between May and August at sustainable harvest levels. Soil respirometry trials using the extracts showed no differences in CO_2_ fluxes between the macroalgal species, different harvesting seasons, or correlation with plant biomass. It is therefore likely that macroalgal extracts impact the plant directly and produce minor impact on soil microbiota. Thus, these results support the use of macroalgal fertilizers as a low-cost and environmentally friendly alternative to chemical fertilisers.

## Introduction

Modern agricultural practices aim to implement sustainable food systems to overcome population pressure, climate change, and land degradation (FAO 2009; Morison and Matthews 2016; EPA 2024). By 2050, the world is expected to reach 9.1 billion people and require a 70% increase in food production to sustain itself. In order to keep up with the global food demand, 90% of crop production has to increase in yield and through land intensification, while 10% has to rely on land expansion (FAO 2009). This is challenging due to the loss of ecosystem function and soil fertility, especially by using agrochemicals. The excessive use of chemical fertilisers leads to soil acidification, groundwater contamination, eutrophication of waterways and a range of detrimental effects on human health (EPA 2024). The prolonged use of chemical fertilisers leads to the accumulation of toxins and metals in the soil, which can be absorbed by plants and disrupt their growth and development (Priya et al. 2023). Subsequently, these toxins and metals can spread into agricultural produce, building in concentration within the trophic levels of the food chain; and potentially mixing and accumulating in the environment for long periods of time without breaking down (Nabulo et al. 2011; Shuqin and Fang 2018; Mitra et al. 2022). Agrochemicals disrupt chemical signalling between plants and nitrogen-fixing bacteria, reducing the efficiency of their symbiosis (Fox et al. 2007). Despite these issues, farmers still prefer to use artificial fertilisers because of their convenience, quick release, high elemental availability, and lower dosage requirements than organic fertilisers (Han et al. 2016; Karthik and Jayasri 2023). However, their high costs bring concerns over food security in underprivileged nations since insufficient supply leads to low crop productivity and food shortages (Hebebrand and Laborde 2022). In addition, climate change poses a challenge to farming by increasing the variation in rainfall patterns, affecting occurrences of drought, flooding and water availability for irrigation; with rising sea levels threatening low-lying agricultural areas through significant economic losses, increased soil compaction, and waterlogging (Morison and Matthews 2016). These conditions have led to the search for novel industries that help to meet global food demand and receive economic compensation for its environmental benefits, such as macroalgae aquaculture and their extracts.

Macroalgae have been used for thousands of years as a source of food, medicine, cosmetics, dyes and fertilisers (Battacharyya et al. 2015). The global macroalgae industry is worth €8.1 billion, accounting for harvesting 35.82 million wet tonnes of macroalgae per year and 1,604 dry tonnes per km^2^ of the area cultivated by macroalgae aquaculture (FAO 2021; Scottish_Government 2022). Most macroalgae production is focused in Asia, accounting for 97.38% of global production through macroalgae cultivation (Zhang et al. 2022). In Europe, conversely, macroalgae cultivation is limited to 0.80% of production and instead wild species collection is relied upon (FAO 2022). Despite occupying less than 0.005% of global cultivated areas and less than 0.05% of wild macroalgae habitats, macroalgae cultivation promotes habitat protection and mitigates climate change by potentially sequestrating 1,500 t CO_2_ km^−2^ year^−1^ (Duarte et al. 2017). The steady growth of the macroalgae market highlights the opportunity to boost their potential as plant biostimulants and biofertilisers. Plant biostimulants are substances that improve a plant’s natural processes by enhancing nutrient availability, efficiency, abiotic stress tolerance, and crop quality traits (Du Jardin 2015; Patel et al. 2017; Del Buono 2021). Biofertilisers are composed of natural compounds and living microorganisms that enhance biotic and abiotic properties in the soil, thereby promoting plant growth (Ronga et al. 2019). The industry for seaweed-based plant biostimulants and biofertilisers focus on the content of bioactive compounds found in macroalgae, including macronutrients such as carbohydrates, proteins and lipids, and secondary metabolites such as amino acids, betaines, polysaccharides, phytohormones, phenols and osmoprotectants (Meng et al. 2022; Singh et al. 2025). In addition, macroalgae have beneficial elements such as N, P, K, Ca, Fe, Mg, Na, S and Zn (Rayirath et al. 2009). The overall role of organic and inorganic compounds in plants is to regulate growth, maturation and yield (Patel et al. 2017; Karthik and Jayasri 2023); but macroalgae have also been found to increase soil water holding capacity and tolerance to environmental stress, pests, and diseases (Sheela et al. 2017). Therefore, macroalgae are beneficial to plant growth through multiple routes, leading to their potential as a commercial plant biostimulant.

Macroalgae fertilisers can be made from red, green, and brown macroalgae, but less than 1% of seaweed species are used commercially to make fertilisers, leaving the vast majority of species unexploited for their potential as biostimulants (Karthik and Jayasri 2023). The majority of industrial producers use brown macroalgae due to their larger size and higher proportion of bioactive compounds than other types of macroalgae (El-Beltagi et al. 2022; Ismail et al. 2023), but also for their abundance and wide distribution across tropical to temperate marine and intertidal regions (Battacharyya et al. 2015; Zayed and Ulber 2020). Overall, brown macroalgae have 30-40% polysaccharides and 3-15% protein in dry weight, including alginates, fucoidans, laminarins and glucans (Khan et al. 2009; Rayirath et al. 2009). They also have high concentrations of phenols, which are involved in antioxidant activity, metal chelation and cellular protection (Andjelković et al. 2006; Wang et al. 2009). Due to the large range and variability of bioactive compounds, industries use different techniques to maximise yield, biochemical composition, and their effect on plant growth. These methods are influenced by time, pH, cost, quantity, and targeted compounds (Dulanlebit and Hernani 2023). Most conventional methods use solid-liquid extractions to transfer soluble nutrients into solvents such as water, hexane, ethanol or acetone (Grosso et al. 2015; Quitério et al. 2022). This is done by grinding fresh or dry macroalgae and processing them through aqueous extracts, acid hydrolysis or alkaline hydrolysis (Sible et al. 2021). Commercial seaweed biostimulant products such as Maxicrop and Doff are made of the brown intertidal wrack *Ascophyllum nodosum* while other products such as Kelpak and Dora are made of brown sub-tidal kelp *Ecklonia maxima* (Doff 2025; Dora 2025; Kelpak 2025; Maxicrop 2025). *Ecklonia maxima* grows in the southern hemisphere, especially around the southern regions of Africa (Maneveldt et al. 2024); so was not suitable as a comparator for our study. *Ascophyllum nodosum* is highly prevalent across the North Atlantic Ocean including around the United Kingdom and could be used as a comparative standard for the studies. Among the commercial products, Doff liquid seaweed concentrate was selected for its flexibility, sold as a plant biostimulant that can be mixed with soluble or liquid solvents; and for regular applications during the growing season (Doff 2025).

*Ascophyllum nodosum* has long been considered as a plant biostimulant, due to its relatively high content of antioxidants, betaines, phenols and flavonoids (Mackinnon et al. 2010; Fan et al. 2011; Verma et al. 2021). Other macroalgae such as *Fucus vesiculosus* and *Fucus serratus* also have potential as plant biostimulants and biofertilisers since they have similar distribution, abundance and bioactive compounds, which were previously demonstrated in germination, bioremediation and soil conditioning trials (Oluwadare et al. 2020; Krautforst et al. 2023). However, few trials have considered the impact of seasonal variation on the composition of these species, and even fewer on the impact of this variation on their potential as biostimulants.

Seasonal variation in macroalgae have shown significant changes in the proportion of key molecules such as carbon, nitrogen, ash, carbohydrate cellulose, laminarin, mannitol, alginate, polyphenolics and other proteins and metals (Adams et al. 2011a, 2011b; Schiener et al. 2015). Macroalgae nutrient composition can also vary according to species, location, season and temperature (Khairy and El-Shafay 2013). Macroalgae are adapted to tolerate fluctuations in wave action, photoperiods, salinity, nutrients, temperature, desiccation, ultraviolet radiation and contaminants (Vinuganesh et al. 2022). This adaptation is done by changing metabolic processes, which results in different biotic-abiotic interactions that regulate the uptake of minerals and the biosynthesis of metabolites (Marinho-Soriano et al. 2006). Adaptation to fluctuating conditions leads to variations in biochemical constituents in primary and secondary metabolites; fatty acids, minerals, antioxidants and pigments (Vinuganesh et al. 2022). Light is the strongest factor influencing macroalgal growth since it is directly involved with the concentration and composition of photosynthetic pigments (Schmid et al. 2017). Thus, research on macroalgae seasonal variation often compares nutritional components between summer and winter since light and temperature levels peak during the summer (Sampath-Wiley et al. 2008). For instance, many studies on brown macroalgae reported increased vitamins, minerals and amino acids in the summer (Castro-González et al. 1994; Paiva et al. 2018). However, lower pigment concentrations occurred in brown and red macroalgae during the summer due to a higher ratio of xanthophylls to chlorophylls (Schmid et al. 2017). Other studies found higher protein and carbohydrate levels and lower lipid levels during the summer (Paiva et al. 2018). Therefore, seasonal variation can be used to determine when to harvest macroalgae to make fertilisers, aiming to reduce dependence on chemical fertilisers and offer a more sustainable crop production despite the rise in population and climate change.

Soil microbiota also play a crucial role in maintaining soil health and enhancing plant performance. For instance, soil microbes regulate carbon storage, nutrient cycling, and the decomposition of organic material, which increases nutrient availability and stimulates plant growth (Wang et al. 2024). Their role in the carbon cycle is quantified as the rate at which they respire and release carbon from soils, which depends on temperature, moisture and pH (Wu et al. 2024). It is estimated that photosynthesis fixes 12 Pg C year^−1^ while the autotrophic respiration releases 64 ± 12 Pg C year^−1^ and heterotrophic respiration releases 43.6 ± 19.3 Pg C year^−1^ (Konings et al. 2019). Despite having lower inputs than outputs, the soil still represents the second largest carbon reservoir in the planet, and its long-term storage of carbon can heavily influence climate change on the short-term (Janzen 2004; Davidson and Janssens 2006). Increasing soil carbon is key to reducing greenhouse gas emissions since soil respiration releases ten times more CO_2_ annually than anthropogenic sources (Dutta and Dutta 2016). Thus, current research on the soil microbiota has focused on studying the mechanisms behind CO_2_ fluxes and its potential as a strategy to mitigate global warming (Jat et al. 2022). Other works have studied the impact of macroalgae fertilisers in soil structure and microbial composition (Lanno et al. 2021; Qiqin et al. 2023). A few studies have explored the impact of macroalgae on soil respiration (Haslam and Hopkins 1996), but none were found to examine the influence of macroalgae seasonality.

This study aimed to evaluate the impact of the seasonality through the application of extracts from 12 monthly-harvested *A. nodosum*, *F. serratus*, and *F. vesiculosus.* The study compared the above-ground biomass of model crop *A. thaliana*, *B. vulgaris*, and *L. sativa* seedlings following applications of the extracts at three different concentrations in low-nutrient compost. A second related study compared the CO_2_ flux from sandy loam soil following applications from macroalgae extracts produced during different seasons.

## Methods

### Plant growth

Plant experiments were conducted in a controlled environment growth room equipped with cool white LED lamps (280 µmol photons m^−2^s^−1^) and an upward airflow distribution system. Room temperature was maintained between 20 - 22 °C with an 8 h:16 h light:dark regime. Three crops were selected: *Arabidopsis thaliana* (Col-0, from internal stocks at Aberystwyth University), 'Moneta' beetroot (*Beta vulgaris*; Mr Fothergills, Newmarket, United Kingdom) and 'Tom Thumb' hearting lettuce (*Lactuca sativa*, Mr Fothergills).

*Arabidopsis thaliana* was sown in trays using 80% (v/v) F2 compost (Levington, Cardiff, United Kingdom) and 20% (v/v) horticultural silver sand (RHS, London, United Kingdom). After two weeks of growth, plants were pricked out and transferred into individual 6 cm diameter pots using Bulrush compost (Bulrush Horticulture Ltd, Magherafelt, United Kingdom) containing 0.25% Yara 15-10-20 fertiliser (YaraMila, Oslo, Norway). F2 compost and horticultural silver sand were used for optimal seed sowing of *A. thaliana*, following recommended growth practices. *Lactuca sativa* and *B. vulgaris* seeds were sown directly into 6-cm diameter pots filled with Bulrush compost containing 0.25% Yara fertiliser. Bulrush compost and Yara fertilizer were used to create a low-nutrient substrate with sufficient nutrients to support minimal plant growth, aiming to show that any significant growth could result from the addition of commercial macroalgae biostimulants or macroalgae extracts. Soil characteristics are summarised in Table 1.

**Table 1:** Chemical characteristics of substrates used in growth trials

Nutrient Content	F2 Compost^a^	Bulrush Compost^b^	Yara 15-10-20 Fertiliser^c^
Nitrogen (N)	14%	14%	15%
Phosphorus (P)	7%	12%	10%
Potassium (K)	24%	24%	20%
pH	5.3–5.8	6–8	4–7

^a^ CTS Garden Supplies, (2025); ^b^ Bulrush, (2023); ^c^ Yara, (2025).

### Macroalgae source and extract preparations

*Ascophyllum nodosum*, *Fucus vesiculosus* and *F. serratus* were collected monthly between November 2021 and October 2022 from Aberystwyth shores (52°24'54.7"N 4°05'27.5"W). Samples were frozen, freeze-dried, and ground into fine powder, then stored in zip-lock bags within an opaque container. Macroalgal extracts were prepared following the hot-water extraction following Chi et al. (2018), where a ratio of macroalgae to water is established and boiled with water for 2 h. Macroalgae powder from each species-month (*n* = 108) was placed in 50-mL Falcon tubes (Starlab, Belgium) and mixed them with deionised water in a ratio of 1:20, 1:50 and 1:100 (w/v). Samples were vortexed for 15 s and transferred into a shaking water bath (Isotemp SWB 15, Fisher Scientific, UK) for 2 h at 75 °C, vortexing again every 30 min. Samples were centrifuged at 3,220 ×*g* for 5 min at 4˚C and the supernatant was pipetted into 2-mL microfuge tubes (Greiner, Austria). The remaining supernatant was transferred into 15-mL Falcon tubes (Starlab). Macroalgae extracts were divided into smaller weekly batches and stored with their residues at −20˚C. To ensure extract stability, each batch was defrosted weekly prior to use, avoiding the freezing and thawing of the same stock.

### Application of treatments

Individual plants for each crop were arranged within a randomised layout (*n* = 1053) where every spot was randomly assigned to a treatment of specific macroalgae species and collection month, minimising bias from lighting. Once assigned a position, plants would remain in their spot until the end of the experiment. Three controls were used: tap water, deionised water only and commercial macroalgae fertiliser “Doff Liquid Seaweed” (Doff Portland Ltd, Hucknall, UK). These controls were chosen to reflect a medium that is commonly used to water plants (tap water), a medium without most impurities found in tap water (deionised water) and a commercially-available medium prepared from brown macroalgae (Doff Liquid Seaweed), which can be easily compared as a benchmark. The recommended dilution rate for Doff Liquid Seaweed biostimulant was used (1:300). All plants were additionally watered regularly with tap water for the duration of the experiment to maintain soil moisture.

Macroalgae extracts and control treatments were applied after one month of growth to each seedling type, with each trial lasting three weeks and treatments applied weekly (days 0, 7, 14 and 21). For each application, 200 μL treatment solution was injected onto the soil per pot using disposable 1 mL syringes without needles. Weekly batches of macroalgae extracts were defrosted at 5˚C 12 - 24 h prior to application.

### Plant harvests and weight assessments

On day 22 of each trial, the above-ground biomass for each crop was harvested and placed into individual labelled envelopes. Samples were dried in an oven at 70 °C for at least three days, then weighed to calculate dry mass.

### Soil respiration

Soil was collected from the University of Reading research farm at Sonning, Berkshire, UK (latitude 51°28'53.7"N, longitude 0°53'48.8"W). The soil was sieved to 4 mm and stored in a sealed container in a 4 °C fridge with sub-samples removed and used for characterisation as summarised in Table 2 (Kehoe and Sizmur, pers. comm., 2024).

**Table 2:** Physiochemical properties of arable soil from Sonning, Berkshire, UK

Physicochemical properties	Value
Texture	Sandy loam
Organic matter (%)	2.58 ± 0.06
Sand (%)	64.27 ± 1.13
Silt (%)	34.29 ± 0.91
Clay (%)	1.11 ± 0.32
% water holding capacity	30.88
pH	6.09 ± 0.03
Dissolved organic carbon (mg kg^−1^)	51.47 ± 1.59

In this experiment, 1:100 dilutions were selected for soil respirometry based on the results from the biomass assay. An equivalent of 10 g (dry weight) of soil was amended with 1 mL macroalgae extract and 400 μL deionised water or 1.4 mL deionised water as the comparative control treatment (n = 45). Each soil sample was gently mixed after adding the solutions and stored in a sealed capsule. Samples were sealed as soon as possible without standardisation since substrate-induced respiration can only be fully quantified by capturing the period after substrate addition until the respiration rate stabilises. Once all the capsules were sealed, these were transferred into an automated multichannel respirometer and a multi-sample gas exchange system (EGA60, ADC Bioscientific Ltd, UK) to continuously measure CO_2_ flux using infrared spectroscopy at hourly intervals for 110 h within a closed, temperature-controlled room set to 26 °C. Two EGA60 multichannel respirometer instruments were employed simultaneously to undertake the measurements, each with 25 channels, including one channel that contains a soda lime CO_2_ scrubber to ‘zero’ the instrument at the start of an experiment. Each channel has its own diaphragm pump to ensure that CO_2_-free ambient air was circulated at a constant rate through each sealed capsule at a rate of 200 µmol s^−1^. The instrument was programmed so that the air flowing out of each capsule in turn was directed to the infrared detector for 150 s, during which the concentration of CO_2_ in the air was measured for 30 s (allowing time before measurement to flush the previous sample out) before moving on to the next capsule. Once a measurement has been made on all 24 capsules, the instrument returns to the first capsule and repeats the process for all 24 analytical capsules, resulting in one CO_2_-flux measurement made each hour (150 s multiplied by 24 analytical channels = 60 min).

### Data Analysis

Statistical tests were performed to compare results against three controls: tap water, deionised water and the commercially available Doff macroalgae fertiliser. Independent samples t-tests were used in seedling dry weight analysis through SPSS (IBM SPSS Statistics, 29.01.1), assuming that significant variance between groups were present. Initial manipulations and graphical representations performed in Excel 365 (Microsoft, Version 2309, Build 16811.20004) and python (Python, 3.12.2). Where seedlings died within the growth trials (7/351 beetroot seedlings and 1/351 lettuce), an average was generated from the remaining duplicates for the triplicate value. Two-way ANOVAs were conducted to analyse mixed effects and interactions between control types, crop types, macroalgae species, macroalgae collection month and macroalgae extract concentrations. Instrument malfunction resulted in erroneous CO_2_ flux data for half of the individual capsules and the data associated with these capsules was removed from the dataset. Because this issue occurred randomly across the dataset, there was a mixture of *n* = 0, *n* = 1, *n* = 2, and *n* = 3 for individual experimental treatments. Remaining samples were compiled and normalised in Excel 365 (Microsoft) against deionised water control to show CO_2_ flux for each month at each time point, with each composed of an average CO_2_ flux of *A. nodosum*, *F. serratus* and *F. vesiculosus*. The CO_2_ flux from each sample was analysed against control conditions (no treatment) through independent samples t-test. One month representing mid-spring (April), one month representing mid-summer (July), one month representing mid-autumn (October), and one month representing mid-winter (January) was identified where a dataset of sufficient size was obtained (*n* = 3) in the majority of cases (*n* = 2 in 3 cases). The CO_2_ flux for these months was analysed against control conditions (no treatment) through one-way ANOVAs, assuming that samples were independent, and variances were homogeneous. Where only duplicates were available, an average was generated for the triplicate value.

## Results

Seedling dry weights were compiled and analysed according to the macroalgae extract concentration, macroalgae species and month of macroalgae collection for every crop. A two-way ANOVA revealed clear differences in dry weight between all crops (*p <* 0.001), but no difference between controls in either crop; and no interaction between them (Table 3). A second two-way ANOVA revealed significant differences in independent variables (*p <* 0.001) including macroalgae species (*p <* 0.01), month of macroalgae collection (*p <* 0.01) and crop type (*p <* 0.001). Post-Hoc tests showed that treatments with *A. nodosum* extracts yielded the lowest dry weight and treatments with *F. serratus* and *F. vesiculosus* extracts were statistically equal. Treatments with macroalgae extracts from January had the highest significant dry weight while macroalgae extracts from July had the lowest dry weight. *L. sativa* had the highest dry weight while *A. thaliana* and *B. vulgaris* were statistically equal. There were significant interactions between macroalgae month of macroalgae collection*macroalgae species (*p <* 0.01), macroalgae species*extract concentration (*p <* 0.05), macroalgae species*crop type (*p <* 0.001), month of macroalgae collection*macroalgae species*extract concentration (*p <* 0.001) and macroalgae species*extract concentration*crop type (*p <* 0.001). Individual data points were used for subsequent analyses.

**Table 3:** Two-way ANOVA comparing the significant differences and interactions between macroalgae species, month of macroalgae collection and crop type for the average plant dry weight. All significant differences were positive until prefixed by a negative sign (-)

**Significance (*p*)**	
**Source**	***P***
Month	**
Seaweed	**
Concentration	
Crop	***
Month * Seaweed	*
Month * Concentration	
Month * Crop	
Seaweed * Concentration	*
Seaweed * Crop	**
Concentration * Crop	
Month * Seaweed * Concentration	***
Month * Seaweed * Crop	
Month * Concentration * Crop	
Seaweed * Concentration * Crop	***
Month * Seaweed * Concentration * Crop	

^*^= <0.05 significance, ** = <0.01 significance and *** = <0.001 significance.

### *Arabidopsis thaliana* dry weight

**Fig. 1:**
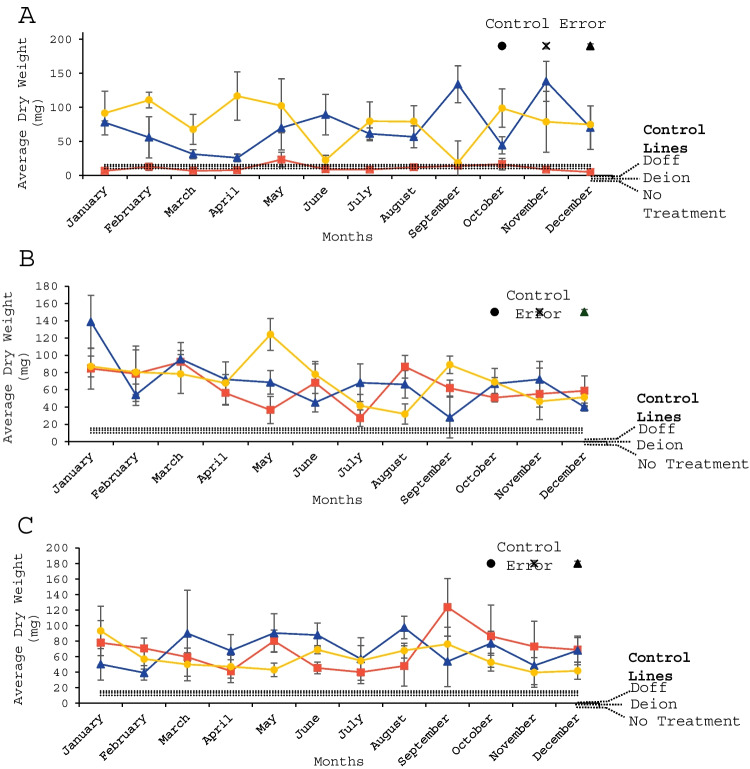
Average dry weight (mg) of *A. thaliana* plants grown after amendment with (**A**) 1:20 extracts, (**B**) 1:50 extracts and (**C**) 1:100 extracts of *A. nodosum* (red squares), *F. serratus* (blue triangles) and *F. vesiculosus* (yellow circles) collected and extracted during different months of the year, compared to controls: no treatment, deionised water (Deion) and Doff fertilizer (Doff). *n* = 3 for each data point presented except controls, where *n* = 9. Error bars show standard error, where black circle = no treatment, black cross = deionised water and black triangle = Doff fertiliser

All controls had highly similar results, thus the highest control (Doff fertiliser) was selected and used as the comparative control against the macroalgae-treated plants (Fig. 1 and Table 4). For 1:20 dilutions there was a minimal growth effect from *A. nodosum* with lower *A. thaliana* weight than the Doff fertiliser controls across many harvest months. The other two macroalgae (*F. serratus* and *F. vesiculosus*) produced significantly greater *A. thaliana* weight when amended with extracts of macroalgae collected in January (*p* < 0.001), March (*p* < 0.01) and December (*p* < 0.001). The 1:50 dilutions showed significantly greater *A. thaliana* weight than the Doff fertiliser control for all macroalgae species collected in April (*p* < 0.001), October (*p* < 0.001) and December (*p* < 0.001). The 1:100 dilutions produced the best results with all macroalgae species resulting in significantly greater *A. thaliana* weight when macroalgae was collected in February (*p* < 0.01), March (*p* < 0.001), May (*p* < 0.001), June (*p* < 0.001) and August (*p* < 0.05).

**Table 4:** Average dry weight of A. thaliana plants grown with 1:20, 1:50 and 1:100 extracts of *A. nodosum, F. serratus and F. vesiculosus* harvested at each month given; compared to Doff fertiliser, the highest-yielding control in this study

**Significance (*p*)**												
	Jan	Feb	March	April	May	June	July	Aug	Sep	Oct	Nov	Dec
**1:20 dilution**												
*A. nodosum*	-*		-*	-*								-**
*F. serratus*	***		**	*			*					
*F. vesiculosus*		*										***
**1:50 dilution**												
*A. nodosum*			*					*		***		
*F. serratus*				***								***
*F. vesiculosus*	*				*	*			*	***	*	
**1:100 dilution**												
*A. nodosum*			***		*	***						
*F. serratus*		**				*		*				
*F. vesiculosus*					***	***		*				

*n* = 3 for each data point presented except Doff fertiliser, where *n* = 9. All significant differences were positive unless prefixed by a negative sign (-) against the highest control (Doff). *= <0.05 significance, ** = <0.01 significance and *** = <0.001 significance.

### Beta vulgaris dry weight

**Fig. 2:**
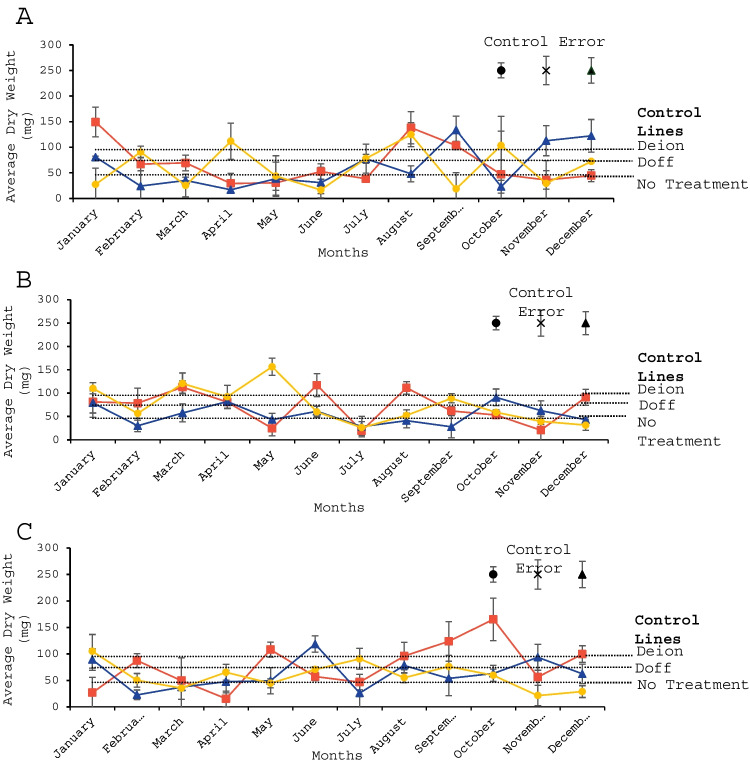
Average dry weight (mg) of *B. vulgaris* plants grown after amendment with (**A**) 1:20 extracts, (**B**) 1:50 extracts and (**C**) 1:100 extracts of *A. nodosum* (red squares), *F. serratus* (blue triangles) and *F. vesiculosus* (yellow circles) collected and extracted during different months of the year, compared to controls: no treatment, deionised water (Deion) and Doff fertilizer (Doff). *n* = 3 for each data point presented except in seven cases, where *n* = 2; and in controls, where *n* = 9. Error bars show standard error, where black circle = no treatment, black cross = deionised water and black triangle = Doff fertiliser

Results across all macroalgae concentrations revealed that deionised water obtained the highest control followed by Doff fertiliser and tap water (Fig. 2). Thus, the deionised water treatment produced highest biomass of the control treatments, followed by Doff fertiliser and tap water (Table 5). For 1:20 dilutions there was a significantly lower *B. vulgaris* weight than deionised water when amended with extracts of *F. serratus* collected in April (*p* < 0.05), *F. vesiculosus* collected in June (*p* < 0.05) and *A. nodosum* collected in July (*p* < 0.05). The 1:50 dilutions only showed significantly lower *B. vulgaris* weight when amended with extracts of *A. nodosum* collected in July (*p* < 0.05).

**Table 5:** Average dry weight of B. vulgaris plants grown with 1:20, 1:50 and 1:100 extracts of *A. nodosum, F. serratus and F. vesiculosus* harvested at each month given; compared to deionised water, the highest-yielding control in this study. *n* = 3 for each data point presented except in seven instances, where n = 2; and for Doff fertiliser, where *n* = 9. All significant differences were positive until prefixed by a negative sign (-) against the highest control (Doff)

**Significance (*p*)**												
	Jan	Feb	March	April	May	June	July	Aug	Sep	Oct	Nov	Dec
**1:20 dilution**												
*A. nodosum*							-*					
*F. serratus*				-*								
*F. vesiculosus*						-*						
**1:50 dilution**							-*					
*A. nodosum*												
*F. serratus*												
*F. vesiculosus*												
**1:100 dilution**												
*A. nodosum*												
*F. serratus*												
*F. vesiculosus*												

^*^= <0.05 significance, ** = <0.01 significance and *** = <0.001 significance.

### Lactuca sativa dry weight

**Fig. 3:**
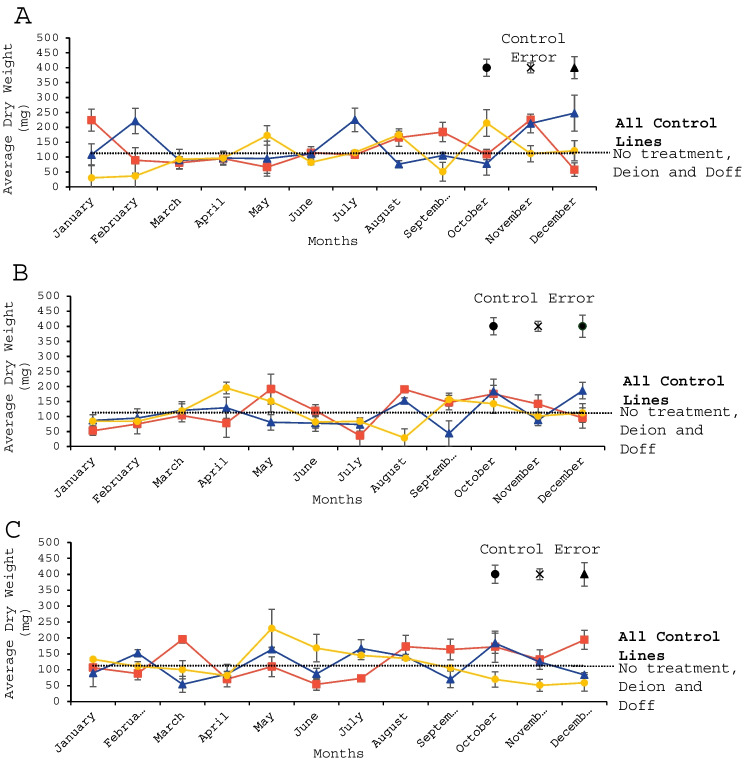
Average dry weight (mg) in *L. sativa* plants grown after amendment with (**A**) 1:20 extracts, (**B**) 1:50 extracts and (**C**) 1:100 extracts of *A. nodosum* (red squares), *F. serratus* (blue triangles) and *F. vesiculosus* (yellow circles) collected and extracted during different months of the year, compared to controls: no treatment, deionised water (Deion) and Doff fertilizer (Doff). All three control average dry weights were highly similar in this study, so appear as one line on the figure. *n* = 3 for each data point presented except in one case, where *n* = 2; and in controls, where *n* = 9. Error bars show standard error, where black circle = no treatment, black cross = deionised water and black triangle = Doff fertiliser

All controls produced similar biomass in *L. sativa*, thus the highest control (Doff fertiliser) was selected and used as the comparative control against the macroalgae-treated plants (Fig. 3 and Table 6). For 1:20 dilutions there was a significantly higher *L. sativa* weight than Doff fertiliser when amended with extracts of *F. vesiculosus* collected in August (*p* < 0.05) and *A. nodosum* collected in November (*p* < 0.05). However, there was a significantly lower *L. sativa* weight when amended with extracts of *F. serratus* collected in April (*p* < 0.05) and *F. vesiculosus* extracts collected in June (*p* < 0.05). The 1:50 dilutions only showed a significantly higher *L. sativa* weight when amended with extracts of *A. nodosum* collected in August (*p* < 0.05).

**Table 6:** Average dry weight of L. sativa plants grown with 1:20, 1:50 and 1:100 extracts of *A. nodosum, F. serratus and F. vesiculosus* harvested at each month given; compared to no treatment, the highest-yielding control in this study. *n* = 3 for each data point presented except in one instance, where *n* = 2; and for Doff fertiliser, where *n* = 9. All significant differences were positive until prefixed by a negative sign (-) against the highest control (Doff)

**Significance (*p*)**												
	Jan	Feb	March	April	May	June	July	Aug	Sep	Oct	Nov	Dec
**1:20 dilution**												
*A. nodosum*											*	
*F. serratus*				-*								
*F. vesiculosus*						-*		*				
**1:50 dilution**												
*A. nodosum*								*				
*F. serratus*												
*F. vesiculosus*												
**1:100 dilution**												
*A. nodosum*												
*F. serratus*												
*F. vesiculosus*												

^*^= <0.05 significance, ** = <0.01 significance and *** = <0.001 significance.

### Significant interactions by crop type

Since multiple interaction effects were found, the data was split by crop type to conduct additional two-way ANOVAs (Table 7). There were only significant differences in variables for *A. thaliana* (*p <* 0.001). Results revealed significant differences in macroalgae species (*p <* 0.001), extract concentration (*p <* 0.001) and their interaction (*p <* 0.001). Post-Hoc tests for *A. thaliana* showed that *A. nodosum* extracts gave the lowest dry weight while *F. serratus* and *F. vesiculosus* were statistically equal. Other post-Hoc tests showed that 1:50 extracts produced the highest yield while 1:20 extracts produced the lowest yield and 1:100 extracts were not significant (*p <* 0.001).

**Table 7:** Two-way ANOVA comparing the significant differences and interactions between macroalgae species, month of macroalgae collection and extract concentration for the average *A. thaliana* dry weight. All significant differences were positive until prefixed by a negative sign (-)

**Significance (*p*)**	
Source	*P*
Month	
Seaweed	***
Concentration	*
Month * Seaweed	
Month * Concentration	
Seaweed * Concentration	***
Month * Seaweed * Concentration	

^*^ = <0.05 significance, ** = <0.01 significance and *** = <0.001 significance.

### Number of significant increases and decreases for all crops

**Fig. 4:**
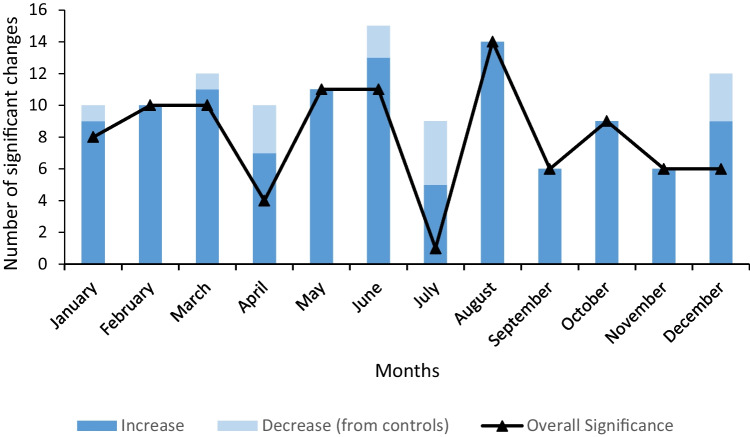
The number of significant increases and decreases in average plant biomass dry weights across all crops compared against all controls for macroalgae collected in each month. The overall significance line is shown as a bar graph across each month based on a +1 value to represent an individual significant increase and −1 value to represent an individual significant increase for each case (*n* = 39)

The number of significant increases and decreases in plant biomass across all crops was compared against each relevant highest seedling control weight, according to macroalgae extract seasonality (Fig. 4). Results were also compiled into an overall significant effect line graph, where the number of significant weight decreases in treated seedlings from control seedlings was subtracted from the number of significant seedling weight increases for each harvest month (*n* = 39). Macroalgae harvested in August and June had the highest number of weight increases in seedlings and overall weight increases in the seedlings than when macroalgae harvested in other months was applied.

### Soil respirometry

**Fig. 5:**
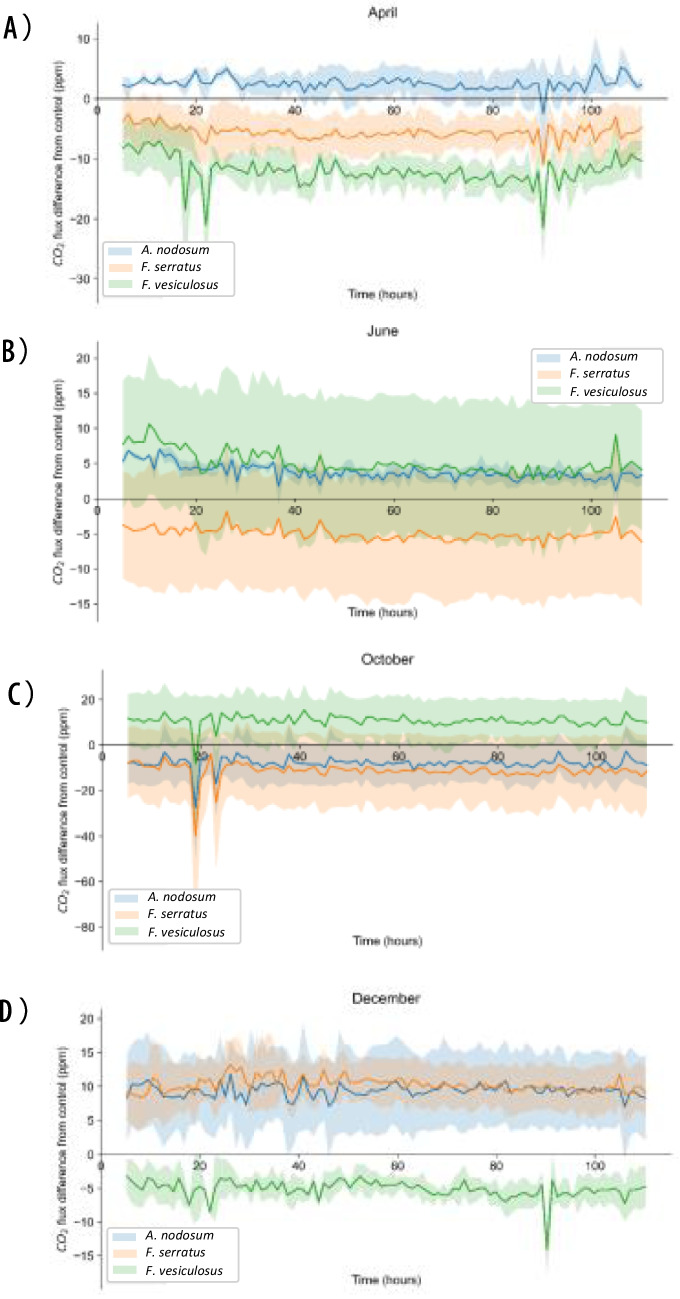
CO_2_ flux (ppm) difference between deionised water (x-axis) and seaweed-treated samples (*n* = 36) harvested in (**A**) April (Spring), (**B**) June (Summer), (**C**) October (Autumn) and (D) December (Winter). Blue line = *A. nodosum*, orange line = *F. serratus* and green line = *F. vesiculosus*. The standard error is shown by the individual shaded areas.

Soils mixed with macroalgal extracts were analysed to provide data on soil respiration. Fig. 5 shows the average CO_2_ flux across seasons, using deionised water as the x-axis and each macroalgae species as individual lines. One-way ANOVAs were performed to compare results at comparable timepoints between 5 - 110 h (e.g. 5 h with 5 h, 6 h with 6 h). There was a significant difference in CO_2_ flux between soils amended with macroalgae collected during different seasons at 19 h (*p* < 0.01) and 23 h (*p* < 0.05). At 19 h, November had a significantly lower CO_2_ flux than April (*p* < 0.05), June (*p* < 0.01), September (*p* < 0.01) and December (*p* < 0.01). November also had a significantly lower CO_2_ flux than June (*p* < 0.05) and December (*p* < 0.05) at 23 h. Due to the high frequency of these measurements and the significance of these time points, a timeframe from 16 - 26 h was selected to conduct further analyses. Despite the previous results, individual samples t-tests revealed no significance between each macroalgae-treated sample and its respective control (Doff fertiliser for *A. thaliana*, deionised water for *B. vulgaris* and Doff fertiliser for *L. sativa*).

## Discussion

Growth assays for *A. thaliana*, *B. vulgaris* and *L. sativa* revealed that most weight increases occurred when treated with more diluted macroalgae extracts. For instance, most plant biomass increases were identified at 1:100 dilutions in *A. thaliana* and 1:50 dilutions in *L. sativa*. No visible extract-induced differences were observed in *B. vulgaris*, but this could be due to the large seed size of this crop, which may provide more nutrients during initial growth stages than smaller seeds (Ambika et al. 2014). The results suggested that dilute dosages of all macroalgae extracts could induce better growth responses in seedlings than the most concentrated dosage additions. This effect can also be observed in macroalgae and chemical fertiliser growth assays. Studies on *Brassica rapa* with *E. maxima* extracts in 1:1 and 1:2 dilutions concluded that reducing the nutrient solution concentration by at least 50% did not affect yield or biomass (Di Stasio et al. 2017). The same effect occurred in growth assays on hydroponic *L. sativa* with chemical nutrient solutions in 1:1, 1:10, and 1:100 dilutions (Chen et al. 1997). Higher fertiliser concentrations can lead to lower plant performance. Growth assays in *Triticum aestivum* seedlings using *Ulva linza* and *Corallina officinalis* extracts in 1:5, 1:10, 1:20, 3:10, and 3:20 dilutions showed that higher macroalgae concentrations led to adverse effects on plant growth (Hamouda et al. 2022). These effects could originate from high concentrations of naturally occurring compounds and plant hormones in macroalgae extracts such as polyphenols, ABA, auxin and cytokinins (Aina et al. 2022; Moncada et al. 2022; Smith et al. 2024). The excessive accumulation of growth regulating compounds and their interaction with soluble sugars, amino acids and minerals can activate plant hormone signalling (Spence and Bais 2015; Silva et al. 2019), altering gene expression and inducing metabolic changes that disrupt plant growth (Ghaderiardakani et al. 2019; Kergosien et al. 2023). Dilution results can be misleading when compared to other studies since they use different starting concentrations, allowing variable aqueous contents for initial and subsequent dilutions. For instance, a similar study used 1:100 dilutions as a starting extraction and considered this a 100% concentrated filtrate before diluting them into 1:5, 1:10, 1:20, 3:10, and 3:20 dilutions (Hamouda et al. 2022). When compared to this study, these extracts would equate to 1:500, 1:1000, 1:2000 and 3:2000 dilutions, significantly more diluted than in this trial (Hamouda et al. 2022). Nevertheless, the study correlates that less concentrated dosages are better than more concentrated dosages despite the variability in starting dilutions found in previous studies.

The rate of nutrient uptake may depend on crop species and ecotypes (Di Stasio et al. 2017). In this study, the environmental conditions were kept constant, thus differences in plant metabolism could lead to different rates of nutrient uptake, especially since seed size could impact plant performance and the absorption of macroalgae extracts (Ambika et al. 2014). *Beta vulgaris* seeds were much bigger than *A. thaliana* and *L. sativa* seeds, which could impact seedling growth. Larger seeds have larger energy reserves, and seedlings with more energy stores could rely on them longer than smaller seeds (Naylor 2003; Ambika et al. 2014). The opposite can be observed in smaller seeds, especially in *A. thaliana* where the plant focuses its energy reserves to stimulate initial growth and build photosynthetic tissues (Réthoré et al. 2019). Since environmental factors in the growth chamber were kept constant, it is suggested that differences in plant traits and plant biomass allocation can both influence growth rates and strategies. For instance, large-seeded species across many plant families invest more energy to build below-ground biomass than short-seeded species (Lloret et al. 1999; Simpson et al. 2021). This would apply to *B. vulgaris* since they have big seeds and develop fleshy taproots at the base of the stem during the first growing season and only focus on developing the seed stalk in the second year (Mall et al. 2020; CFIA 2023). Germination in *A. thaliana* and *L. sativa* was faster than in *B. vulgaris*, a trend frequently observed in smaller seeds (Souza and Fagundes 2014). Based on the short duration of the experiment, most of the nutrients absorbed in *B. vulgaris* could have originated from the seed’s energy reserves instead of the soil and the macroalgae treatments, which would explain the lack of significant trends between 1:20, 1:50 and 1:100 dilutions compared to the other crops. Since the initial stages of germination relies on the seed’s energy reserves, it is unlikely that the difference in substrates used for germination in *A. thaliana* and *B. vulgaris* could have influenced differences in the results (Bewley and Nonogaki 2017). Despite this, seasonal variability can regulate the production and concentration of these compounds and influence the effectiveness of the biostimulant effects.

Peaks of greater plant biomass were observed with fertilisers made from macroalgae collected in February, May, August and October. These results can be correlated to nutrient and fertility peaks, which can influence the best harvest date for fertiliser use. Studies on the seasonal variation of polysaccharides in *A. nodosum*, *F. serratus* and *F. vesiculosus* revealed a gradual build-up of fucoidan contents from spring to autumn, peaking in late autumn and decreasing in winter (Fletcher et al. 2017). There is an increase in mineral, amino acid, phenolic, β-carotene and tocopherol contents in brown macroalgae throughout summer (Castro-González et al. 1994; Paiva et al. 2018). Despite having limited data on the collection dates for fertiliser industries, brown macroalgae can be harvested all year round in the United States and United Kingdom, with a preferred harvest date from March to August (MSC 2013). These dates also correlate to peaks of greatest plant growth and peaks in fertility between May-June for *A. nodosum* and *F. vesiculosus* and August-October for *F. serratus* (MSC 2013*)*. Since brown macroalgae release spores and propagules from the tip of fronds, it can be recommended to harvest *A. nodosum*, *F. serratus* and *F. vesiculosus* for fertiliser use between May and August when there are nutrient and fertility peaks.

*Arabidopsis thaliana* showed the most cases of enhanced plant growth compared to its control (Doff fertiliser), with 27 cases of increased biomass, most pronounced in plants treated with macroalgae extracts harvested in March, June and August and slightly decreasing in plants treated with macroalgae extracts harvested from September to January; but showing an overall greater biomass compared to untreated plants. Since all crops were grown for the same amount of time, the increased number of greater biomass cases for *A. thaliana* can be attributed to *A. thaliana*’s fast growth cycle, especially since *A. thaliana* can complete its life cycle within six weeks while *B. vulgaris* takes 12 weeks and *L. sativa* takes 10-14 weeks (Masson 2001; Collins 2024; RHS 2024). *Lactuca sativa* and *B. vulgaris* shared a similar number of increased biomass cases, focusing on samples produced from spring-summer and late autumn-early winter collections. However, *B. vulgaris* and *L. sativa* had very few cases of significant biomass changes when compared to their respective controls (Doff fertiliser or deionised water). *Beta vulgaris* only had biomass decreases when compared to deionised water while most biomass changes in *L. sativa* resulted in weight increases when compared to Doff fertiliser, especially with extracts made from macroalgae collected in August. Despite many reports on increased macronutrients and metabolites in macroalgae during the summer, these results indicate that some crops benefit more from macroalgae fertilisers than others (Paiva et al. 2018). In this case, *A. thaliana* benefited the most from macroalgae extracts, which could result from a greater absorption of macroalgae-derived nutrients instead of the nutrient reserves inside their seeds. This can be attributed through *A. thaliana*’s small seed size, which has minimum nutrient reserves that are only sufficient to support initial embryo growth and the early development of photosynthetic tissues (Réthoré et al. 2019). Nevertheless, an extended growth trial in *B. vulgaris* and *L. sativa* could reveal if the results are limited by nutrient seasonality or differences in growing stages.

Multiple interaction effects could have influenced differences in plant growth for each crop. The lack of significant statistical differences between controls and their interactions with crop types suggests that seedlings from any crop did not react differently to controls. Based on the significant interactions between independent variables, macroalgae species was the most interactive factor since it was linked to all independent factors. Interactions between macroalgae species and month of macroalgae collection suggest that the bioactivity of macroalgae extracts depends on the type of macroalgae and the seasonal variation of their nutrients (MSC 2013; Fletcher et al. 2017). Despite that, the highest dry weight was not found in within the recommended harvest date and nutrient or fertility peaks for either macroalgae species, results showed that macroalgae can be harvested in any month as long as the sustainability regulations are met and environmental conditions are optimal (MSC 2013; Scottish Marine Assessment 2020). The interaction between macroalgae species and extract concentration is influenced by extraction method and the dilution used throughout the study (Cikoš et al. 2018). Since the study used the same concentrations and extraction methods for all macroalgae, any differences in plant growth can be attributed to the inhibitory effects of highly concentrated extracts and the seasonal variation in bioactive compounds in macroalgae (MSC 2013; Fletcher et al. 2017; Silva et al. 2019; Kergosien et al. 2023). Crop type influenced the results more than the month of macroalgae collection, macroalgae species and extract concentrations. The majority of the variation in the data came from *A. thaliana* since it was the only crop to have significant differences in independent variables and interactions between macroalgae species and extract concentrations. This variation could be due to differences in seed size and biomass allocation compared to other crops (Bewley and Nonogaki 2017). Furthermore, comparisons between biomass assays and respirometry studies can reveal seasonal trendlines for each macroalgae.

Respirometry data showed no correlation with the seasonal differences seen in the plant growth trials, especially when considering the variance in CO_2_ flux for the extracts from each macroalgae and harvest month. For instance, biomass trendlines for 1:100 dilutions showed that *A. thaliana* grows best with extracts from *F. vesiculosus* harvested in May, June and August; but its best results across all dilutions were obtained with 1:50 dilutions using extracts from *F. vesiculosus* harvested in September and October. *Beta vulgaris* and *L. sativa* did not have biomass trendlines or a best macroalgae fertiliser to use based on dilutions and macroalgae species. Respirometry results showed no significant differences in CO_2_ peaks between each season, since CO_2_ flux in response to most macroalgae extracts were constantly above or below the control. However, results occasionally showed low points at 20 and 90 hours after the extract was mixed with the soil. These results reflect disagreement in the literature since previous studies have found significant increases in soil respiration after adding fertilisers (Peng et al. 2020; Huang et al. 2021), but other studies have reported the opposite effect (Fang et al. 2017; Wang et al. 2019), or no effect at all (Chen et al. 2017). Other studies reported higher soil respiration rates when the soil was amended with 8.2 g kelp kg^−1^ compared to unamended controls, but lower respiration rates than those produced by amending 8.2 g kelp kg^−1^ were obtained by amending 16.4 g kelp kg^−1^ into the soil (Haslam and Hopkins 1996). Despite sharing similar macroalgae concentrations, the results from this experiment contrasts with the ones of the aforementioned study since the current study used liquid extracts instead of solid fertilisers, which have a higher bioavailability of nutrients, uniform consistency and a faster absorption by plants (Izydorczyk et al. 2022). Solid fertilisers tend to have more carbon than liquid fertilisers since they have a higher proportion of elements and organic matter contained in a solid form, which are gradually released over time (Regelink et al. 2021).

Studies investigating the effect of macroalgae seasonality on soil respiration have not yet been reported in the literature. Thus, more research is needed to explore the relationship between macroalgae seasonality and its impact in soil microbial communities when applied as an extract. Regardless of this, the rise in CO_2_ for macroalgae-treated samples can be attributed to the depletion of macroalgae nutrients added to the soil, which results in the acceleration of microbial respiration and growth (Walker et al. 2018; Zhou et al. 2021).

In conclusion, the present studies showed the effects of harvesting month and dilution magnitude of *A. nodosum*, *F. serratus* and *F. vesiculosus* extracts on *A. thaliana*, *B. vulgaris* and *L. sativa* growth. Results showed that macroalgae extract-treated plants had greater dry weights than those without treatment, deionised water, or commercial macroalgae extract Doff fertiliser. Among the macroalgae concentrations trialled from concentrated initial preparations, 1:50 dilutions on *A. thaliana* were most effective for plant growth compared to their highest control, in contrast to the other crops since no optimum fertiliser dilutions were observed. Despite this, variability in *B. vulgaris* growth yields suggests that as anticipated, different plants have varied metabolisms and nutrient requirements, suggesting that that an extended growth trial would allow more time for biomass allocation. There was greater biomass in plants treated with extracts from June and August, which correlates with fertility peaks in these macroalgae throughout the summer. Based on these observations, it is proposed that the optimal harvest time for these macroalgae for plant growth enhancement is between May and August in the Northern Hemisphere. Due to the lack of an influence of macroalgae on CO_2_ flux in the soil respirometry trials, it is likely that nutrients from macroalgae fertilisers have minor impact on the soil microbial activity. Therefore, the extracts are likely to impact the plant directly, but this assumption warrants further experimental trials. Overall, these results encourage the use of macroalgae fertilisers, offering a low-cost and environmentally friendly alternative to artificial fertilisers.

## Data Availability

Raw and statistical data in excel form has been deposited in Mendeley data, (https:/data.mendeley.com/datasets/ctytmbbn29/1)
